# Nucleotide mismatches between the *VP7 *gene and the primer are associated with genotyping failure of a specific lineage from G1 rotavirus strains

**DOI:** 10.1186/1743-422X-3-35

**Published:** 2006-05-25

**Authors:** Gabriel I Parra, Emilio E Espinola

**Affiliations:** 1 Departamento de Biología Molecular, Instituto de Investigaciones en Ciencias de la Salud, Universidad Nacional de Asunción. Río de la Plata y Lagerenza, Asunción (2511), Paraguay

## Abstract

In recent years it was reported that the accumulation of point mutations in *VP4 *and *VP7 *genes of rotavirus strains was the main cause of the failure of the G or P-typing. Failures in the correct genotyping of G1, G2, G8, G9 and G10 rotavirus strains were reported in the most commonly used reverse transcription (RT)-PCR strategies. Collecting *VP7 *gene sequences of G1 rotavirus strains from databases we found that 74 (61.2 %) out of 121 G1 strains from lineage I showed the four specific mismatches at the 5' end of the 9T1-1 primer, previously associated with the failure of G1-typing. Thus, a great percentage of the G1 strains from lineage I worldwide reported could not have been typed if the Das's RT-PCR strategy were used. This analysis shows that the failure on the detection of the G1 strains could be due to the diversification of rotavirus strains in phylogenetic lineages. Therefore, the use of different RT-PCR strategies with different primer binding locations on the *VP7 *gene or new typing methodologies -like microarrays procedures- could be a better option to avoid the failure of the G-typing of rotavirus strains detected during surveillance programs.

## Findings

Causing more than 450,000 deaths per year, group A rotaviruses are the most important cause of acute diarrhea in children throughout the world [1].

Based on the antigenicity and amino acid differences from the two outermost proteins, VP4 and VP7 respectively, group A rotaviruses are classified into P and G-types [2]. At the time, at least 26 P and 15 G-types were detected [2-4], most of them showing a high degree of intragenic diversification due to point mutations, insertions and/or deletions [5-12]. Although the most frequent human G-types of a given geographical region change from season to season, the genotype G1 is considered the most prevalent worldwide [13].

Since the vaccination against rotavirus may induce selective effects on the diversity of strains, vaccine-escape mutants could emerge. In order to evaluate the vaccine efficiency, the surveillance programs should detect the diversity of rotavirus strains before, during and after the introduction of a rotavirus vaccine [14].

Since the introduction of reverse transcription (RT)-PCR for rotavirus genotyping, many epidemiological surveillances have been conducted and new data has been collected to understand this complex epidemiology [15]. However, in recent years it was reported that the accumulation of point mutations in *VP4 *and *VP7 *genes was the main cause of the failure of the G or P-typing of rotavirus strains [7,8,12,16-21].

At least, there are four multiplex RT-PCR strategies commonly used for rotavirus G-typing [17,22-24] and one for P-typing [25]. In the one developed by Gouvea et al [24], it was reported failures in the correct genotyping of G2, G8, G9 and G10 rotavirus strains [7,12,17,20,21], and recently it was suggested the use of modified or degenerated primers to avoid the mismatches between the primer and the *VP7 *gene [17,20].

In a recent paper published by Rahman et al [19], it was reported the failure of the Das's RT-PCR strategy to detect most (75%) of the G1 human rotavirus isolated in Bangladesh during the surveillance in 2002. They argue that this failure was due to four mismatches found at the 5' end of the primer binding site. Although two G1 strains correctly typed had a 100% identity with the untypeable strains, they concluded that the remainder 25% could be typed because the 3' end of the primer binding site had a perfect match.

In order to evaluate how many G1 strains included in the GenBank database have these four specific mismatches and its clustering within specific lineages, we collected 173 sequences of the *VP7 *gene from G1 strains from the GenBank database Release 151, December 2005 (alignments are available from the authors on request). A phylogenetic tree was constructed from aligned coding sequences, using Neighbor-joining or parsimony methods with Kimura 2-parameter as a model of nucleotide substitution with the MEGA 3.1 [26] and Phylip v3.65 softwares. The statistical significance of the tree was preformed by bootstrapping, using 1000 pseudo-replicates data sets.

**Figure 1:**
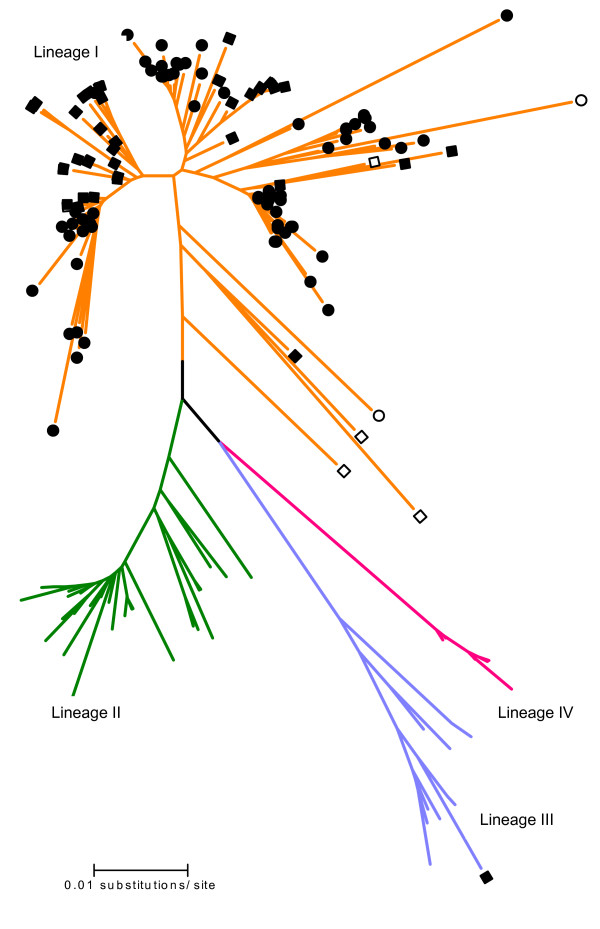
Phylogenetic tree showing the four lineages described in genotype G1 of rotaviruses. The strains having the four mutations reported by Rahman and his colleagues are indicated by ●; the four mutations plus others at the primer binding site by ○; three out of the four mutations by ■, and three mutations plus others at the primer binding site by □. The open branches indicate one or two mutations at the primer binding site. The lineages are represented in the tree as follows: lineage I (orange), lineage II (green), lineage III (blue) and lineage IV (red).

All the strains grouped within one of the four lineages previously reported by Jin et al [5] (Fig. 1). The typeable and untypeable G1 strains reported by Rahman et al [19], grouped within the lineage I with a high bootstrap value (95 %) (data not shown). Seventy four (61.2 %) out of 121 G1 strains from lineage I showed the four specific mismatches at the 5' end of the 9T1-1 primer. This was associated with the failure of genotyping of G1 strains from Bangladesh. Two of them showed more than four nucleotide mismatches. Forty two (34.7 %) showed only 3 out of the four nucleotide mismatches and the remainder 4.1% showed 3 out of the four nucleotide mismatches plus others mismatches at the 9T1-1 primer binding site. The strains grouped within lineage II, III and IV, showed no more than two nucleotide mismatches, except for one sample from lineage III (Brz-2; GenBank number: U26362) that showed three out of the four nucleotide mismatches (Fig. 1).

Interestingly, when we compared the 173 sequences of G1 strains with the aBT1 primer binding site, we found that 170 strains showed one or two nucleotide mismatches and 3 showed three mismatches, suggesting that Gouvea's RT-PCR strategy could type these strains correctly (data not shown).

Thus, taking into account that 75% of the G1 strains with four nucleotide mismatches were not detected during the surveillance in Bangladesh [19], probably a high percentage out of the 121 strains from lineage I, included in our analysis, could not be typed by the Das's RT-PCR strategy. It is noteworthy that G1 strains from lineage I were the most reported worldwide.

It was suggested to use modified or degenerated primers, or change the priming binding site, in order to avoid the mismatches between the primer and the *VP7 *gene [7,17,20]. However, the failure of the G-typing of rotavirus strains, detected during surveillance programs, could be avoided through different RT-PCR strategies that use different primer binding locations on the *VP7 *gene [7,19,27] or by new typing methodologies, like microarrays procedures [28,29].

This analysis shows that the failure on the detection of the G1 strains could be due to the diversification of rotavirus strains in phylogenetic lineages, as it was showed by Santos et al [7] in G9 strains when they used the Gouvea's RT-PCR strategy.

## Authors' contributions

GIP performed the sequences analysis, phylogenetic analysis and wrote the manuscript; EEE download the sequences from database, performed the sequences analysis and critically revised the manuscript.
